# The Tm7sf2 Gene Deficiency Protects Mice against Endotoxin-Induced Acute Kidney Injury

**DOI:** 10.1371/journal.pone.0141885

**Published:** 2015-11-05

**Authors:** Leonardo Gatticchi, Ilaria Bellezza, Rachele Del Sordo, Matthew J. Peirce, Angelo Sidoni, Rita Roberti, Alba Minelli

**Affiliations:** Department of Experimental Medicine, University of Perugia, Piazzale Gambuli, 06124 Perugia, Italy; National Institutes of Health, UNITED STATES

## Abstract

Cholesterol is essential for diverse cellular functions and cellular and whole-body cholesterol homeostasis is highly controlled. Cholesterol can also influence cellular susceptibility to injury. The connection between cholesterol metabolism and inflammation is exemplified by the Tm7sf2 gene, the absence of which reveals an essential role in cholesterol biosynthesis under stress conditions but also results in an inflammatory phenotype, i.e. NF-κB activation and TNFα up-regulation. Here, by using Tm7sf2^+/+^and Tm7sf2^−/−^ mice, we investigated whether the Tm7sf2 gene, through its role in cholesterol biosynthesis under stress conditions, is involved in the renal failure induced by the administration of LPS. We found that the loss of Tm7sf2 gene results in significantly reduced blood urea nitrogen levels accompanied by decreased renal inflammatory response and neutral lipid accumulation. The increased expression of fatty acids catabolic enzymes reduces the need of the renal autophagy, a known crucial nutrient-sensing pathway in lipid metabolism. Moreover, we observed that the Tm7sf2 insufficiency is responsible for the inhibition of the NF-κB signalling thus dampening the inflammatory response and leading to a reduced renal damage. These results suggest a pivotal role for Tm7sf2 in renal inflammatory and lipotoxic response under endotoxemic conditions.

## Introduction

Sepsis is a complex disease arising from the host response to an overwhelming infection. Gram-negative bacteria and the components of their walls, in particular, the lipid A-containing lipopolysaccharide (LPS), play a major role in the pathogenesis of sepsis. As in Gram-negative sepsis, during endotoxemia, LPS induces uncontrolled cytokine release, activation of coagulation on endothelial cells leading to shock, multiple organ damage, and even death. During sepsis and endotoxemia, acute kidney injury is a frequent complication [1]. In all mammalian cells, cholesterol is essential for diverse cellular functions. Cholesterol is required for maintenance of membrane fluidity and permeability, regulation of integral membrane protein function, transcriptional regulation, and for lipid raft formation. Therefore, cellular and whole-body cholesterol homeostasis is highly controlled and maintained through a network of transcriptional programs. Indeed, the cycling between cholesterol/cholesterol esters and cholesterol movement between the cellular organelles and the extracellular compartment underscore the complexity of cholesterol homeostasis under physiological, and likely, pathophysiological states [2–3]. Cholesterol can also influence cellular susceptibility to injury. The dramatic increase in cholesterol content that follows the induction of experimental acute renal failure characterises the acquired cytoresistance response, i.e. the partial renal resistance to further ischemic or toxic damage [4–5]. Cholesterol, as a component of lipid rafts, plays also a pivotal role in the recruitment and concentration of molecules involved in cellular signalling triggered by LPS and/or tumour necrosis factor α (TNFα) [6]. TNFα is a pro-death and pro-inflammatory cytokine, which, as one of the multiple factors present in acute kidney injury, actively participates in the pathological process [7–8]. However, studies with TNFα-deficient mice challenged with tunicamycin, an ER stress-inducing agent, clearly showed that TNFα may have a direct role in the renal protection against acute ER stress and that severe injury is associated with extensive accumulations of neutral lipids [9].

We have previously shown that the Tm7sf2 gene plays an essential role in cholesterol biosynthesis under stress conditions and that Tm7sf2 knockout mice have lower levels of hepatic cholesterol after tunicamycin treatment [10]. We also showed that the Tm7sf2 gene directs an anti-inflammatory loop and its absence correlates with an inflammatory phenotype, characterised by NF-κB activation and TNFα up-regulation. The absence of the Tm7sf2 gene hinders the feedback loop to inflammatory stimuli and over expression of hTm7sf2 protein reverses the inflammatory phenotype [10]. Moreover, we recently demonstrated that the loss of Tm7sf2 alters the expression of proteins involved in epidermal differentiation by reducing the levels of cholesterol sulfate and accelerating skin papilloma formation [11]. Because of the complex transcriptional regulatory network connecting cholesterol/lipid homeostasis and inflammation, we hypothesised that: i) these effects of Tm7sf2 gene deficiency might be causally linked; ii) defects in cholesterol biosynthesis might in turn disrupt the finely tuned regulation of the transcriptional factors involved in the inflammatory response.

To further investigate this connection and because our previous data linked Tm7sf2 expression to renal TNF expression [10], a key feature of renal pathology in sepsis [7–8], here we investigated the effects of Tm7sf2 gene insufficiency in a model of experimental endotoxemia and kidney failure induced by the administration of LPS.

## Materials and Methods

### Materials

All the reagents, unless otherwise stated, were from Sigma Aldrich (St. Luis, MO). All the antibodies are listed in Table 1.

**Table 1:** List of antibodies.

Antibody	Application	Dilution	Source
β-actin (C11)	WB	1:400	Santa Cruz Biotechnology
Iba-1 (sc-1022-5)	IHC	1:100	Santa Cruz Biotechnology
iNOS (M-19)	WB	1:400	Santa Cruz Biotechnology
p-p65 (p-NF-κB; Ser 536)	WB	1:1000	Cell Signaling
Caspase 3	WB	1:1000	Cell Signaling
LC3B	WB	1:2000	Sigma-Aldrich
Cyt c (sc-13156)	WB	1:500	Santa Cruz Biotechnology
COX-IV (sc-58348)	WB	1:2000	Santa Cruz Biotechnology
GAPDH (sc-32233)	WB	1:500	Santa Cruz Biotechnology

WB—Western blotting; IHC—Immunohistochemistry.

### Animals

C57BL/6 Tm7sf2^+/+^ (WT) and Tm7sf2^−/−^ (KO) mice (>20 backcross) [12] were housed at the Laboratory Animal Research Centre of Perugia University. The animals were maintained at a constant temperature of 22°C, 12h light/dark cycle, and fed ad libitum.

### Ethics Statement

All experimental procedures were carried out in accordance with European Directives, approved by the Institutional Animal Care and Use Committee of Perugia University (106/2012). Efforts were made to minimise animal stress/discomfort.

### Animal treatment

Male C57BL/6J, Tm7sf2^−/−^ (n = 20), and Tm7sf2^+/+^ (n = 20) mice were intraperitoneally injected with a single dose of LPS (5mg/kg) or vehicle (0.9% saline). Mice were sacrificed at selected time points and tissues used for mRNA, protein, or histological analyses.

### Laboratory data

BUN (blood urea nitrogen, mg/dl) and creatinine (mg/dl) in plasma were determined using a Hitachi 911 Clinical Chemistry Analyzer (RocheDiagnostics, Indianapolis, IN) by standard methods in the Department of Veterinary Medicine at the University of Perugia (Italy).

### Real-time RT-PCR

Total RNA was isolated with TRIZOL Reagent (Invitrogen Srl, Milano, Italy) according to the manufacturer’s instructions and cDNA was synthesised using iScript cDNA synthesis kit (Bio-Rad Lab, Hercules, CA). PCR reaction products were fractionated through 2% agarose gel stained with 0.5mg/ml ethidium bromide and observed with an UV transilluminator. Real time PCR was performed using the iCycler iQ detection system (Bio-Rad Lab, Hercules, CA) and SYBR Green chemistry. Murine primers were obtained from Sigma-Aldrich. Primers are listed in Table 2. SYBR Green RT-PCR amplifications were carried out in a 96-well plate in a 25μl reaction volume that contained 12,5μl of SYBR^®^ Green JumpStart™ Taq ReadyMix™, 400nM forward and reverse primers, and 5 to 40 ng of cDNA. In each assay, no-template controls were included and each sample was run in triplicates. Mean of Ct values of the samples was compared to the untreated control sample and GAPDH used as internal control. The n-fold differential ratio was expressed as 2^-ΔΔCt^.

**Table 2:** List of primers.

Gene name	Gene	Primer sequence (5’ to 3’)
	symbol	(F: Forward; R: Reverse)
ATP-binding cassette transporter 1	ABCA1	F: CCAGACGGAGCCGGAAGGGT
		R: GTGCCCATGTCCTCGGGAGC
Acyl-Coenzyme A oxidase	ACOX	F: GAATTTGGCATCGCAGACCC
		R: ACGGGTGCATCCATTTCTCC
Carnitine palmitoyl transferase 1	CPT1	F: CATGTATGCCCGCAAACTGG
		R: CCTGGGATGCGTGTAGTGTT
Glycerhaldeyde 3-phosphate dehydrogenase	GAPDH	F: GCCAAATTCAACGGCACAGT
		R: AGATGGTGATGGGCTTCCC
HMGC-CoA reductase	HMGCR	F: TGCCTGGATGGGAAGGAGTA
		R: GCCTCGAGTCATCCCATCTG
Inducible NO synthase	iNOS	F: GTGTTCTTTGCTTCCATGCTAAT
		R: GTCCCTGGCTAGTGCTTCAGA
LDL receptor	LDLR	F: GGGAACATTTCGGGGTCTGT
		R: AGTCTTCTGCTGCAACTCCG
Medium chain acyl-Coenzyme A dehydrogenase	MCAD	F: TCAAGATCGCAATGGGTGCT
		R: GCTCCACTAGCAAGCTTTCCA
Transmembrane 7 superfamily member 2	Tm7sf2	F: GCCTCGGTTCCTTTGACTTC
		R: CCATTGACCAGCCACATAGC
Tumor necrosis factor-α	TNFα	F: GCCCACGTCGTAGCAAACCAC
		R: GGCTGGCACCACTAGTTGGTTGT

### Immunohistochemical analysis

Paraffin-embedded sections from mouse kidney were collected for immunohistochemical (IHC) analysis. Briefly, tissue samples were fixed in 10% buffered formalin and embedded in paraffin. The 4μm tissue sections were stained with hematoxylin & eosin. Frozen sections were used for Oil red O staining to determine the renal accumulation of neutral fats. The IHC was performed on 4μm sections using a mouse monoclonal antibody against Iba-1 (clone 1022–5; 1:100). The primary antibody was detected using a biotin-free polymeric horseradish peroxidase (HRP)-linker antibody conjugate system (Bond Polymer Refine Detection, Vision BioSystems Ltd, Aus) with a heat-induced epitope retrieval, conducted with the Bond-III automated immunostainer (Leica BioSystems Melbourne Pty Ltd, Aus), and mounted for microscopic evaluation (Leica DMD108 digital microimaging). Appropriate negative and positive control slides were carried out in parallel.

### Western Blotting

Tissues (10% w/v) were lysed in boiling Laemmli sample buffer or subjected to mitochondria purification as previously described [13]. Total protein samples were electrophoresed on SDS-polyacrylamide gels and transferred to nitrocellulose membranes. Membranes were probed with the indicated antibodies (Table 1), which were detected using HRP-based chemiluminescence (ECL, Pierce Biotechnology, Rockford, IL). Each sample was prepared identically from five individual animals and, within each group, results were highly reproducible. Each blot was analysed by ImageJ (NIH, http://imagej.nih.gov/ij/).

### Kidney lipids and cholesterol assessment

Renal cortex (20 mg) was homogenized in 19 volumes of PBS plus protease inhibitor cocktail and 1mM PMSF. Lipids were extracted by the Folch method [14] and separated by TLC using n-hexane: ethyl ether: acetic acid (70:30:1, v/v) for cholesterol and triglycerides and n-hexane: ethyl ether: acetic acid (90:10:1, v/v) for cholesterol esters. After staining with Cu-acetate [15] images were acquired using the VersaDoc Imaging System and signals were quantified using Quantity One software (Bio-Rad, Milan, Italy). Authentic standards were run on the same plate to construct calibration curves.

### Statistical analysis

All results were confirmed in at least three separate experiments and expressed as mean±S.D. Unpaired Student’s *t* test was used to analyze differences between two groups. *P*-values<0.05 were considered significant.

## Results

### Tm7sf2 deficiency does not influences cholesterol homeostasis in the endotoxemic condition

**Fig 1:**
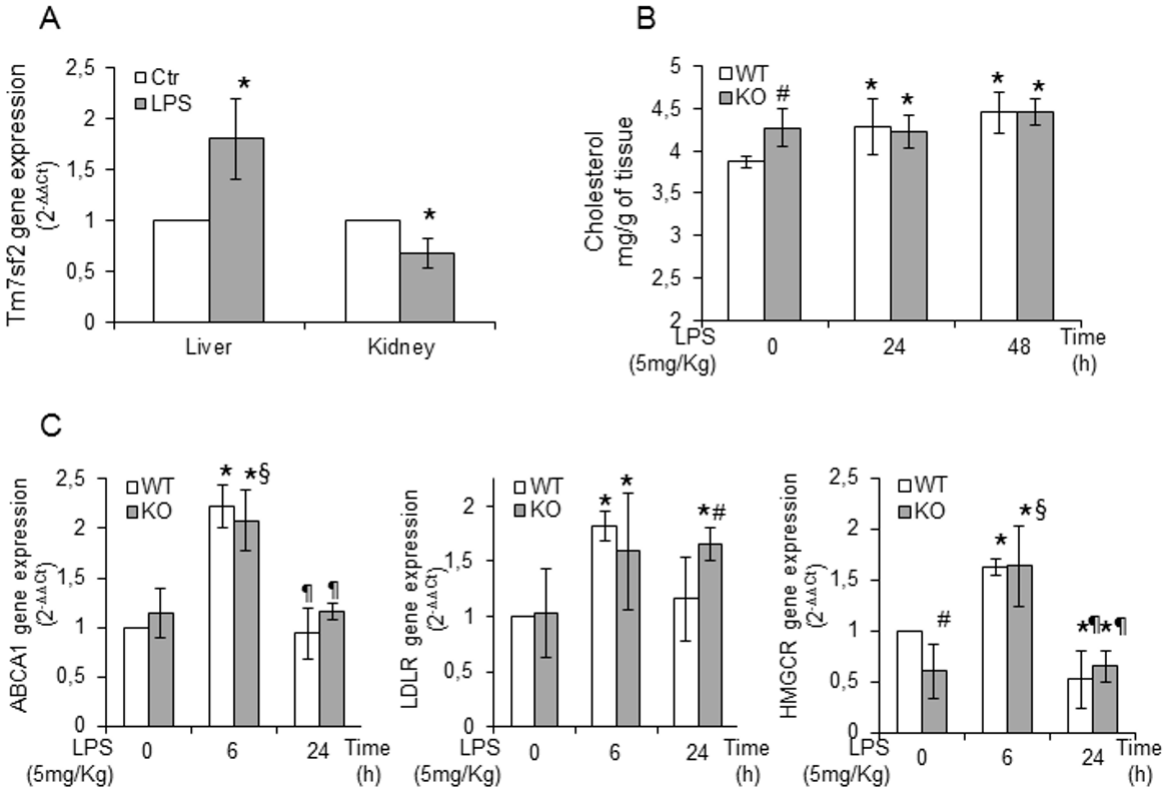
Tm7sf2 insufficiency does not influences cholesterol homeostasis in the endotoxemic condition. (A) Expression of Tm7sf2 gene in Tm7sf2^+/+^ (WT) liver and renal cortex of mice at 3 and 6h LPS-exposure, respectively. (B) Assessment of renal cortex cholesterol levels in 24 and 48h LPS treated WT and Tm7sf2^-/-^ (KO) mice, by TLC. (C) Determination of mRNA expression values of cholesterol homeostasis responsive genes: ABCA1, LDLR and HMGCR by real time PCR analysis. Expression of the genes was normalized to GAPDH and reported as 2^-ΔΔCt^. Relative mRNA level of pooled WT untreated mice (n = 5) was assumed as 1. Results are given as mean ±s.d., (n = 5). *p<0.05 vs. control WT, # p<0.05 vs. the respective WT, § p<0.05 vs. control KO, ¶ p<0.05 vs. the respective 6h.

To investigate the effects of Tm7sf2 gene insufficiency in controlling renal injury in a model of experimental endotoxemia, we used Tm7sf2 WT and KO mice, injected intraperitoneally with 5mg/kg LPS, a relatively low and non-lethal dose that significantly decreases glomerular filtration rate and renal plasma flow, contributing to the induction of acute renal failure [16–17]. The enrichment of renal cholesterol content after an *in vivo* renal injury may be a critical determinant or modulator of acute tubular cell damage [2–5, 18–19]. Therefore, we assumed that the loss of the Tm7sf2 gene, hitherto primarily implicated in cholesterol biosynthesis, might underlie an impaired cholesterol homeostasis/metabolism resulting in an altered response to renal LPS-injury. First, we assessed the role of Tm7sf2 in the response to LPS. At 3h LPS exposure, liver Tm7sf2 mRNA levels were markedly up-regulated while, at 6h LPS exposure, kidney Tm7sf2 mRNA levels were significantly down-regulated (Fig 1A), indicating a different organ response and a different timing of the Tm7sf2 gene to endotoxemic conditions. Next, we determined the cholesterol content in kidneys of mice of both genotypes and found that the renal basal cholesterol levels were more elevated in KO mice, but, at 24/48h LPS exposure, cholesterol levels were similar in both genotypes (Fig 1B). Tissue cholesterol increments can stem from increased up-take via low-density lipoprotein (LDL) receptors, decreased efflux, or de novo synthesis [2]. We determined by real time PCR the expression of the genes responsible for cholesterol level regulation (Fig 1C). Expression of the gene controlling cholesterol efflux, i.e. ABCA1, markedly up-regulated at 6h, reverted to control level at 24h independent of the genotype. Expression of LDLR, responsible for cholesterol up-take, was also significantly up-regulated at 6h in both genotypes, but, at 24h LPS exposure, mRNA levels reverted to control values in WT mice while remaining elevated in KO mice. Finally, expression of HMGCoAR, the rate-controlling enzyme of cholesterol biosynthesis, was markedly and significantly up-regulated at 6h in both genotypes. At 24h, the levels of mRNA reverted to basal values in KO whereas it was down-regulated in WT. However, the mRNA levels of the HMGCoAR gene were similar in both genotypes after LPS exposure, although, under basal conditions, KO mice showed a reduction of 50% in the mRNA levels. Results, showing that the levels of mRNA are independent of murine genotype, indicate that Tm7sf2 gene does not influence renal cholesterol metabolism in endotoxemic conditions.

### Tm7sf2 deficiency reduces the renal inflammatory effects of LPS

**Fig 2:**
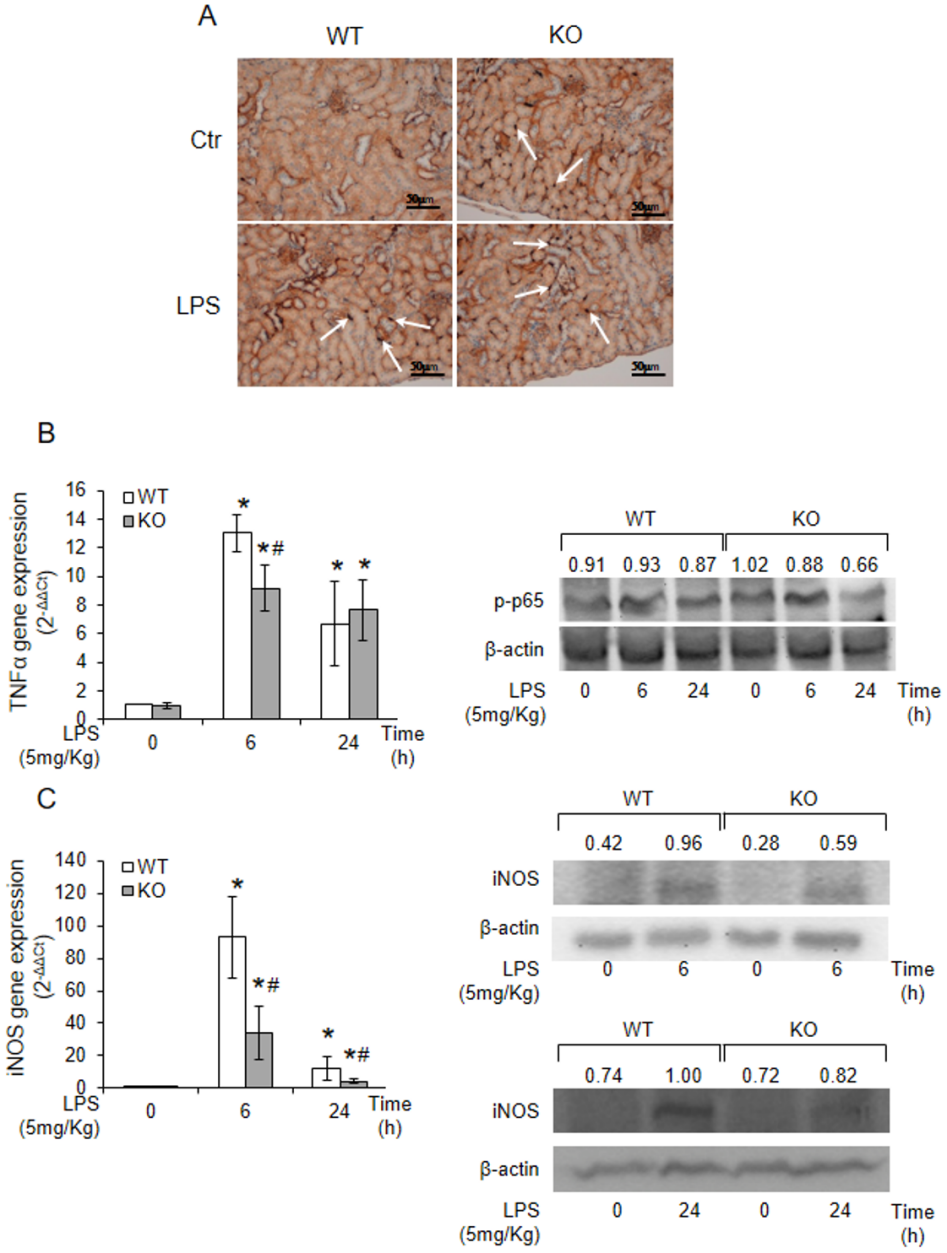
Tm7sf2 insufficiency reduces the renal inflammatory effects of LPS. (A) Iba-1 staining by immunohistochemistry on kidney sections of 48h LPS-treated WT and KO mice. Images magnification, x40. Arrows indicate Iba-1 positive macrophages.(B) TNFα mRNA levels and NF-κB activation were assessed in renal cortex, after 6 and 24 h LPS treatment, by real time PCR and western blotting analysis, respectively. (C) iNOS mRNA/protein levels in kidney cortex after 6 and 24h of LPS-exposure. β-actin antibody was used as loading control. Expression of the genes was normalized to GAPDH and reported as 2^-ΔΔCt^. Relative mRNA level of pooled WT untreated mice (n = 5) was assumed as 1. Results are given as mean ±s.d., (n = 5). *p<0.05 vs. control WT, # p<0.05 vs. the respective WT.

Endotoxemia induced by LPS causes a systemic release of inflammatory cytokines and iNOS-mediated NO generation [8, 20–21]. In addition, experimental data suggest that TNFα can exert controversial roles [22] and in the kidney, TNFα may be directly involved in renal injury [23] or in renal protection [5, 9]. To assess whether inflammation and TNFα could underlie a different response of the two genotypes to LPS, we stained for the presence of ionized calcium-binding adaptor molecule 1 (Iba-1), a marker of activated macrophages, by immunohistochemistry. Kidneys from KO mice were characterised by intense Iba-1 immunoreactivity either in the presence or in the absence of LPS (Fig 2A), whereas WT kidney, showing a moderate immunohistochemical reactivity in the absence of LPS, were as positive as the null genotype at 48h LPS exposure. In the kidney, TNFα expression was also markedly up-regulated by LPS treatment in both genotypes but in the WT kidney, at 6h LPS exposure, it was significantly higher than in KO genotype. At 24h, TNFα expression was similar in both genotypes (Fig 2B). Next, we determined the renal protein expression levels of activated NF-κB and found lower levels of the phosphorylated form in the null genotype (Fig 2B). The reduced activation of NF-κB in the kidney of null mice leads to low iNOS mRNA levels and protein expression in the kidney of Tm7sf2 null mice compared to controls (Fig 2C). Results suggest that the insufficiency of Tm7sf2 can attenuate the inflammatory NF-κB-mediated pathway and the LPS-induced up-regulation of iNOS and subsequent NO production responsible for renal proximal tubule damage [16].

### Tm7sf2 deficiency reduces neutral lipids accumulation in kidney

Lipid accumulation contributes to chronic kidney injury, lipid droplets are dynamic and functionally active organelles involved in lipid metabolism, cell signalling, and inflammation [24]. In renal injury, there is a rise in cortical/proximal tubule cholesterol levels that alters cholesterol/lipid homeostasis [2, 5, 18, 25–27]. Thus, we decided to investigate whether the loss of the Tm7sf2 gene modifies the lipid homeostasis.

**Fig 3:**
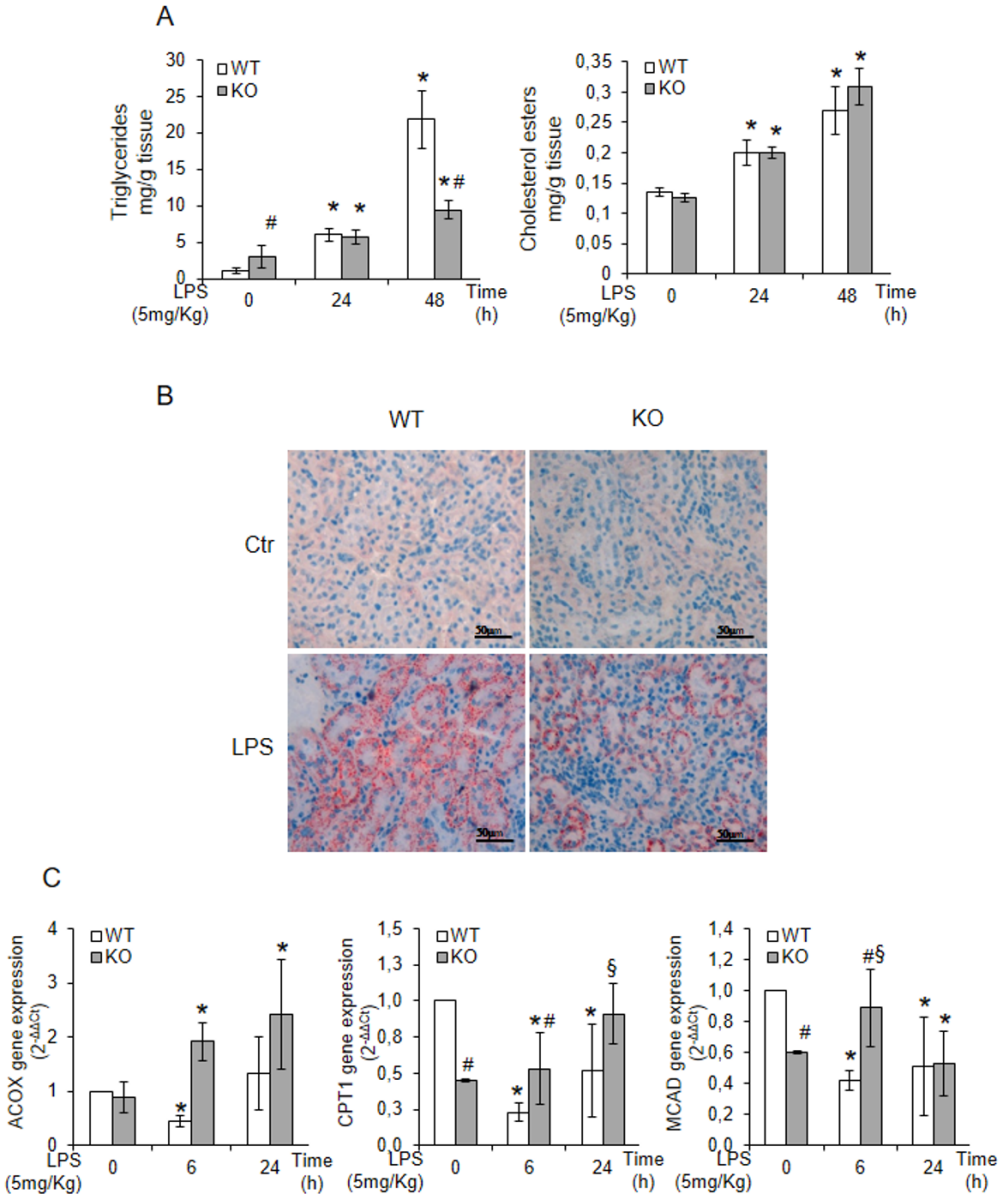
Tm7sf2 insufficiency reduces neutral lipids accumulation in kidney. (A) TLC assessment of triglycerides and cholesterol esters levels in kidney cortex of LPS treated WT and KO mice. Results are given as mean ±s.d., (n = 5). *p<0.05 vs. control WT, # p<0.05 vs. the respective WT. (B) Oil red O staining (ORO) of kidney cryosections. (C) Determination of mRNA expression values of lipid catabolism genes: ACOX, CPT1 and MACD by real time PCR analysis Expression of the genes was normalized to GAPDH and reported as 2^-ΔΔCt^. Relative mRNA level of pooled WT untreated mice (n = 5) was assumed as 1. Results are given as mean ±s.d., (n = 5). *p<0.05 vs. control WT, # p<0.05 vs. the respective WT, § p<0.05 vs. control KO.

Triglyceride levels, although significantly higher in KO mice at basal conditions, were markedly increased by LPS treatment in WT genotype while the levels of cholesterol esters, comparable at basal conditions, were increased by LPS independently of the genotype (Fig 3A). Renal sections, stained with Oil Red O, showed a punctuate staining of neutral lipids less intense in KO mice (Fig 3B), indicating that LPS exposure induces an important accumulation of neutral lipids only in the WT genotype. To investigate whether a decreased degradation underlies the observed lipid accumulation, we analysed the effects of LPS treatment on the transcription of genes involved in lipid catabolism. We found that the genes of the catabolizing enzymes, such as CPT1 (carnitine palmitoyl-transferase I), MCAD (medium-chain acyl-CoA dehydrogenase), and ACOX (acyl-CoA oxidase) were significantly up-regulated in the KO mice while down-regulated in WT mice (Fig 3C).

Results suggest that the presence of the Tm7sf2 reduces lipid catabolism and might be, at least partially, responsible for lipid accumulation that, in turn, can contribute to renal injury.

### Mice deficient in Tm7sf2 gene are resistant to endotoxin-induced renal failure

Because the insufficiency of Tm7sf2 caused a reduction in LPS-induced lipid accumulation and iNOS up-regulation, we surmised that KO mice might be less prone to the kidney failure induced by the administration of LPS.

**Fig 4:**
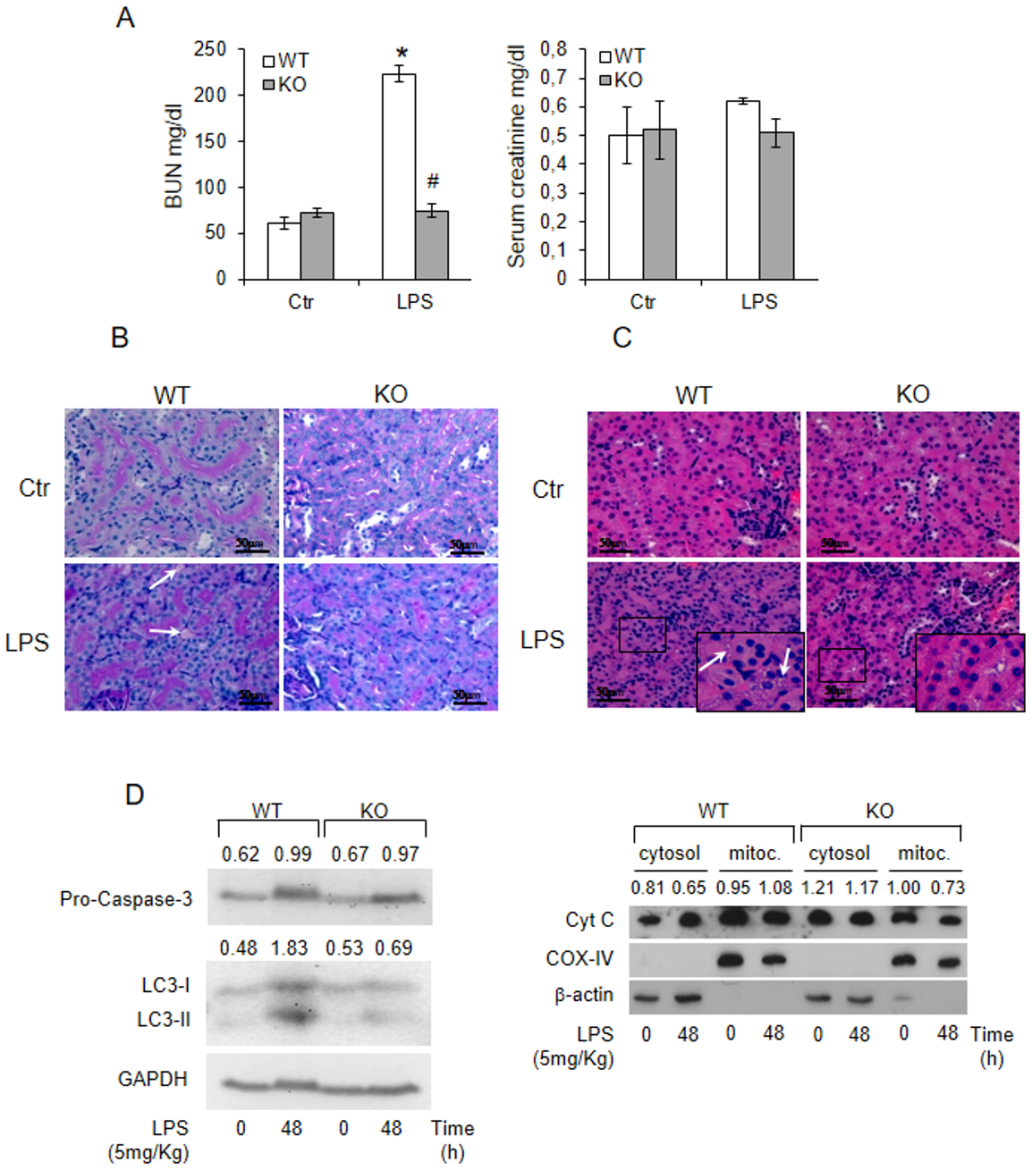
The lack of Tm7sf2 gene protects from LPS-induced acute kidney injury. (A) WT and KO mice were i.p. injected with 5mg/Kg LPS and blood urea nitrogen (BUN) and creatinine were assessed after 24h. Results are given as mean ±s.d., (n = 5). *p<0.05 vs. control WT, # p<0.05 vs. the respective WT. Kidney sections of 72h LPS-treated WT and KO mice were stained with (B) periodic-reactive schiff (PAS) or with (C) hematoxylin/eosin staining. Arrows indicate interstitial oedema and vacuoles. Images magnification, x40.(D) Caspase 3, Cytocrome C, and LC3B in kidney after 48h of LPS-exposure. β-actin and GAPDH antibodies were used as loading control for total and cytosolic extracts. COX-IV was used as loading control for mitochondrial preparation.

We found that blood urea nitrogen (BUN) and creatinine were increased in WT genotype at 24h LPS-treatment while they were unmodified in KO mice (Fig 4A). On periodic acid-Schiff stained sections, a predominant interstitial disease with interstitial oedema was observed in WT mice while renal organization was still present in KO mice (Fig 4B). On hematoxylin & eosin-stained sections, renal tubular cells of WT mice underwent vacuolar degenerative changes (Fig 4C), undetected in KO genotype. Levels of apoptosis, determined by cytochrome c release and lack of caspase 3 activation, were of the same order of magnitude in both genotypes (Fig 4D). Increased abundance of LC3-II, indicative of autophagy [28] were observed only in WT mice (Fig 4E).

## Discussion

The pathogenesis of sepsis and the associated kidney failure is a highly complex process, involving multiple factors, with roles still to be definitely established. Here we showed, for the first time, that the loss of the Tm7sf2 gene, primarily involved in cholesterol biosynthesis, attenuates the extent of renal injury in a model of experimental endotoxemia and kidney failure, induced by the administration of LPS. We also showed that kidney of null mice lacked of the marked accumulation of neutral lipids, which characterizes the WT genotype. Renal cholesterol accumulation, which represents an integral component of a stress response, is associated with cytoresistance to subsequent insults, while accumulation of triglycerides is associated with sensitization to kidney insults [2–5, 19, 25–26]. After LPS administration, renal cholesterol levels were identical in both genotypes, indicating that, in our experimental model, cholesterol is not the key- player of the endotoxemic response. Accumulation of triglycerides and cholesterol esters in tissues not suited for lipid storage results in chronic cellular dysfunction/injury with deleterious consequences on organ function, i.e. lipotoxicity, which contributes to renal disease [27, 29]. Chronic lipotoxicity also contributes to acute renal failure and progression of chronic kidney disease. Formation of lipid droplets, increased by LPS administration in the kidney of WT mice, represents pooling of excess fatty acids and cholesterol esters and accounts for numerous important associated pathologies [27, 30]. A mutually reinforcing interplay between lipid metabolism and inflammation is also known to exacerbate renal lipotoxicity [27, 31–32]. Following LPS administration, both genotypes showed an early up-regulation of TNFα expression that was significantly higher in WT mice. The more robust TNFα induction correlates strongly with the increase in NF-κB activation, macrophage recruitment, shown by the Iba-1 immunostaining, and iNOS gene/protein expression, each observed in WT genotype. Excessive production of nitric oxide (NO) causes systemic vasodilation, which in turn leads to renal vasoconstriction with sodium and water retention. Furthermore, a local induction of iNOS, which is constitutively expressed in the kidney, may be the cause of peroxynitrite-related tubular injury resulting from local formation of reactive oxygen and nitrogen species during systemic inflammation [33]. Therefore, the diminished production of NO, generated by iNOS [16, 34], and the reduced accumulation of neutral lipids could explain, at least partially, the reduced extent of renal damage, demonstrated by the unaltered levels of plasma BUN, observed in the null genotype. Alterations in renal lipid metabolism are known to play an important role in the pathogenesis and progression of renal disease in type 1 diabetes, as well as in obesity and insulin resistance [35], even though the underlying mechanisms are not fully understood. Several studies have revealed that autophagy contributes to lipid metabolism [36–41]. A recent study showed an effect of dysregulated fatty acid β-oxidation on autophagy. In the proximal tubule cells, characterized by high-energy demand and relatively little glycolytic capacity, β-oxidation is the main pathway of fatty acid catabolism and the major source for renal ATP production [42–43]. Thus, the enhancement of β-oxidation reduced the need of autophagy that is a crucial nutrient-sensing pathway in lipid metabolism [28]. LPS is known to decrease renal fatty acids oxidation [44] and to activate the autophagic process [45–46]. We found a down-regulated expression of acyl-CoA oxidase and key proteins required for oxidation of fatty acids in WT genotype whereas a marked up-regulation of the catabolic enzymes characterized the null genotype. Indeed, we observed that the insufficiency of Tm7sf2 causes a robust transcription of acyl-CoA oxidase whose activity is likely responsible for meeting the energy needs of the kidney, thus reducing the autophagic process. It is to note that the enhanced β-oxidation leads to the production of acetyl –CoA, reported as a phylogenetically conserved inhibitor of starvation-induced and age-associated autophagy [47]. Since autophagy can be involved in either cell death or survival based on the cellular context, it needs further experiments to confirm whether the observed autophagy is a pathologic mechanism that ultimately results in renal damage or an adaptive mechanism that are activated by endotoxin treatment. Moreover, it needs to be determined whether the observed reduction of inflammation in KO mice relates to the cellular energy content. Indeed, reports in the literature [48] indicate a link between cellular energy and inflammation, but in our case, the evidence of a clear relationship still requires further investigations.

### Conclusions

The salient findings of the present study are that the loss of the Tm7sf2 gene alters the complex regulatory program that controls the effects of LPS treatment and, by a mechanism still to be clarified in detail, diminishes NO production and lipotoxicity which, in turn, cause an attenuation of the renal damage under endotoxemic conditions

## Supporting Information

S1 Checklist NC3Rs ARRIVE Guidelines Checklist. (PDF)
